# The Immune Epitope Database and Analysis Resource: From Vision to Blueprint

**DOI:** 10.1371/journal.pbio.0030091

**Published:** 2005-03-15

**Authors:** Bjoern Peters, John Sidney, Phil Bourne, Huynh-Hoa Bui, Soeren Buus, Grace Doh, Ward Fleri, Mitch Kronenberg, Ralph Kubo, Ole Lund, David Nemazee, Julia V Ponomarenko, Muthu Sathiamurthy, Stephen Schoenberger, Scott Stewart, Pamela Surko, Scott Way, Steve Wilson, Alessandro Sette

## Abstract

A planned repository of immune epitope data with associated analysis tools should be a boon to vaccine development

## Introduction

Recent concerns about bioterrorism and emerging diseases have led to a new focus on the development of vaccines and drugs targeting infectious pathogens. An important component of vaccine development is the characterization of immune responses (to vaccination, for example, or following infection in experimental settings) by evaluating the epitopes recognized by antigen-specific receptors of the immune system (antibodies and/or T cell receptors (TCRs)) [1]. In recent years, different groups have followed different approaches to the discovery of immune epitopes, and various assay types have been used to generate data for the purpose of epitope definition or validation. We believe that research in this area could be greatly facilitated by a comprehensive knowledge center: a repository of immune epitope data with associated analysis tools. Our goal is the creation of the Immune Epitope Database and Analysis Resource (IEDB).

The IEDB is sponsored by the National Institute for Allergy and Infectious Diseases (NIAID). It will host data relating to both B cell and T cell epitopes from infectious pathogens, as well as experimental and self-antigens (RTP-NIH-NIAID-DAIT-03/31; www.niaid.nih.gov/contract/archive). Priority will be placed on those epitopes considered to be potential bioterrorism threats, and emerging diseases as defined by NIAID (so-called category A–C pathogens; see: http://www2.niaid.nih.gov/Biodefense/bandc_priority.htm). As a corollary to the IEDB effort, NIAID has also launched a large-scale antibody and T cell epitope discovery program aimed at generating epitope data and analysis resources to be included in the IEDB. Other data sources to be integrated into the IEDB are publications in peer-reviewed journals, published patents or patent applications, and direct submissions from institutions or companies. Everyone who contributes data or analysis resources to the database will be cited, either by authorship or by other acknowledgment of their contributions.

The involvement of the scientific community in the design of the scope and capability of the IEDB will be crucial to the success of the project. The IEDB will be produced in a manner that encourages the incorporation of data and analytical tools derived by research labs at-large. With this paper, we hope to inform the scientific community of our effort and to solicit feedback while the project is still in a design stage. We envision that this resource center will be freely available on the Internet, with a prototype operational in the fourth quarter of 2005. Once the project is online, forms for direct feedback and online submission of data and tools will be provided. Yearly conferences to present data relating to epitope identification and the IEDB itself will be organized, and a newsletter will be published quarterly.

## Defining the Scope of the IEDB

Each scientific approach generates a set of epitope data, specific to itself, which must be integrated into a general representation of epitope information. In a programmatic sense, we believe that selecting data that fit one particular epitope definition or experimental bias is not our prerogative and would be unwise. Rather, we have opted to define a comprehensive, all-inclusive representation of information that separates epitope features into intrinsic and extrinsic features. Intrinsic features are those determined by the sequence and structure of an epitope, while extrinsic features are context-dependent attributes determined by the experimental or natural environment. This immunological perspective will be an organizing principle behind the IEDB.

## Intrinsic Versus Extrinsic Features of an Epitope

At the level of T cell epitopes, intrinsic features included in the IEDB are: the molecular structure of the epitope, the binding affinity for different MHC receptors, and the affinity of MHC/ epitope complexes for TCRs of defined sequence. Likewise, at the level of B cell epitopes, intrinsic features include the epitope's molecular structure and binding affinity for antibody molecules of defined sequence. These features are unequivocally specified and are singularly associated with a given epitope structure or epitope/receptor combination.

Other features—such as immunogenicity, or whether an epitope is naturally processed—are not intrinsically associated with a given molecular structure of an epitope alone, but rather are context-dependent (i.e., extrinsic). Context information includes, for example, the species of the host in which a response was found, the assay utilized to measure responses, and the dose and route of administration. Likewise, the yield of a given epitope following proteasomal cleavage of a complex protein precursor is dictated by the overall sequence of the protein in which the epitope is contained. Also, the T cell and B cell responses to an epitope are heavily influenced by previous exposure of the immune system to the same or a related antigen. Collectively, these examples show that to meaningfully capture the immunogenicity of an epitope, the context in which it occurs must be described as well.

## The IEDB Classes

**Figure 1:**
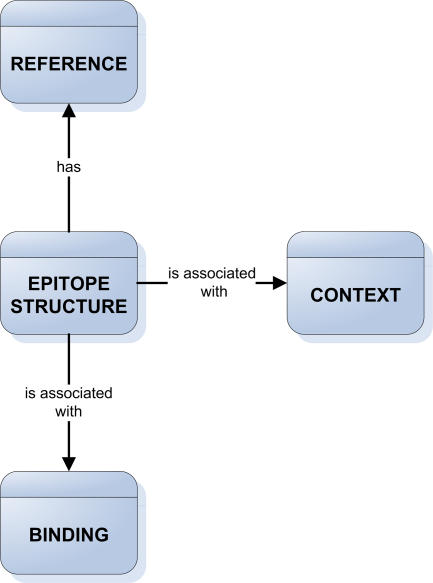
Main Classes in the IEDB

Formalizing the above considerations, we defined the main classes of the IEDB data as Reference, Epitope, Binding, and Context (Figure 1). These classes represent the top level in the data hierarchy used to store epitope information in the IEDB. The class Reference defines one of three possible sources of data, namely literature, patent, and direct submissions. The Epitope class is subdivided into two categories: Epitope Structure, which specifies the molecular structure of an epitope itself, and Epitope Source, which identifies the pathogen/protein in which the Epitope is present. The Binding class captures intrinsic information relating to how the structure specified in the “Epitope” class interacts with well-defined receptors of the immune system such as MHC molecules or antibodies and TCRs of defined sequence. The Context class is organized into three subclasses, including T cell immune responses, naturally processed peptides, and B cell immune responses. Table 1 is an example how the main features of a T cell epitope described in [2] would be displayed in the IEDB. Many more fields exist that are left blank because they are not appropriate for this particular epitope (such as antibody binding data) or are unknown (such as MHC binding data).

**Table 1:**
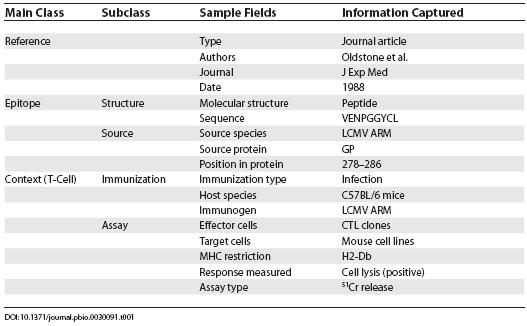
Sample Epitope Entry in the IEDB

## A Scientific Approach for the Development of the Analysis Resource

Our proposal includes the establishment and maintenance of an Analysis Resource of online tools for the Immune Epitope Database. Because this resource must be useful to the entire community, it is important that the tools provided cover a broad range of research areas relating to epitope discovery and analysis, and that no particular scientific “school” has priority. To identify tool candidates, we have generated a list of existing tools of interest through extensive literature searches and expert input. This will be periodically revised, taking advantage of input from the scientific community and NIAID.

The current list of candidate tools comprises an extensive menu of prediction tools for the identification of novel antibody and T cell epitopes from genome and protein sequences. At the level of antibody epitope predictions, standard methods of predicting which regions in a protein are likely to be on the surface will be provided, such as hydrophilicity analysis. Tools that use various methods for prediction of MHC binding will also be provided, along with tools predicting proteasomal processing and TAP transport of T cell epitopes.

We will also provide analytical tool resources to assist in vaccine discovery and development. These are designed to project population coverage of epitopes in different ethnicities, to project the degree of cross-reactivity within sets of different MHC molecules, and to assess the degree of conservancy of an epitope in various isolates of the same pathogen, both in related pathogens, and in potential hosts. Finally, tools to visualize data will be provided, such as those that display antibody antigen interactions where 3D structural information is available. We also hope that collection of consistently annotated data in the IEDB will allow the development of new, “context-sensitive,” tools.

In deciding how many tools should be hosted in the IEDB, a balance has to be achieved between discriminating too much, which may leave user demands unaddressed, and discriminating too little, hosting so many tools that the collection becomes overly redundant and unmanageable. To facilitate an objective and transparent choice of which predictive tools should be hosted, the predictions of all candidate tools will periodically be evaluated. Most importantly, we plan to make all evaluations publicly available through the IEDB website, and we will encourage all different scientific groups to participate by submitting tools and evaluating data. Such prediction “contests” have had a tremendous positive impact in the field of tool evaluation and prediction of protein structure [3,4]. To the best of our knowledge, this would represent the first attempt at a rigorous and comprehensive evaluation of prediction tools found on immune responses.

## Conclusions

We envision a future in which the development of the Immune Epitope Database and Analysis Resource will help researchers throughout the world quickly access relevant information for evaluation of immune responses, assisting them in the development of prophylactic/therapeutic approaches against new and old, emerging and reemerging diseases.

## Abbreviations

IEDB: Immune Epitope Database and Analysis Resource
NIAID: National Institute for Allergy and Infectious Diseases
TCR: T cell receptor
